# Involvement of mTOR in CXCL12 Mediated T Cell Signaling and Migration

**DOI:** 10.1371/journal.pone.0024667

**Published:** 2011-09-12

**Authors:** Rachel Munk, Paritosh Ghosh, Manik C. Ghosh, Takeshi Saito, Mai Xu, Arnell Carter, Fred Indig, Dennis D. Taub, Dan L. Longo

**Affiliations:** 1 Lymphocyte Cell Biology Unit, National Institute on Aging, National Institutes of Health, Baltimore, Maryland, United States of America; 2 Clinical Immunology Section, Laboratory of Molecular Biology and Immunology, National Institute on Aging, National Institutes of Health, Baltimore, Maryland, United States of America; 3 Research Resources Branch, National Institute on Aging, National Institutes of Health, Baltimore, Maryland, United States of America; New York University, United States of America

## Abstract

**Background:**

CXCL12 is a pleiotropic chemokine involved in multiple different processes such as immune regulation, inflammatory responses, and cancer development. CXCL12 is also a potent chemokine involved in chemoattraction of T cells to the site of infection or inflammation. Mammalian target of rapamycin (mTOR) is a serine-threonine kinase that modulates different cellular processes, such as metabolism, nutrient sensing, protein translation, and cell growth. The role of mTOR in CXCL12-mediated resting T cell migration has yet to be elucidated.

**Methodology/Principal Findings:**

Rapamycin, an inhibitor of mTOR, significantly inhibits CXCL12 mediated migration of both primary human resting T cells and human T cell leukemia cell line CEM. p70^S6K1^, an effector molecule of mTOR signaling pathway, was knocked down by shRNA in CEM cells using a lentiviral gene transfer system. Using p70^S6K1^ knock down cells, we demonstrate the role of mTOR signaling in T cell migration both in vitro and in vivo.

**Conclusions:**

Our data demonstrate a new role for mTOR in CXCL12-induced T cell migration, and enrich the current knowledge regarding the clinical use of rapamycin.

## Introduction

Directional motility in response to a chemokine is an inherent property of immune cells. CXCL12 or stromal cell derived factor 1-α (SDF1-α) is a strong chemokine that governs major immune cell migration and trafficking which is orchestrated with membrane remodeling and cytoskeletal rearrangement including actin polymerization [1], [2]. Several distinct signaling pathways are reported to be involved in CXCL12-induced migration including JAK/STAT, PI3 kinase, and MAP kinases [3], [4], [5], [6], [7]. Mammalian target of rapamycin (mTOR) has been shown to be involved in chemokine signaling [8], [9], [10]. mTOR is a serine-threonine kinase that modulates different cellular processes, such as metabolism, nutrient sensing, translation, and cell growth [11], [12]. The contributions of PI3 kinase and mTOR in T cell trafficking has been reported recently [13], [14]. Involvement of mTOR in chemokine mediated migration of T and B cells has been shown recently in hypomorphic mice generated by neo-insertion that partially affects mTOR transcription [15]. In the present study, we wanted to investigate the involvement of mTOR, particularly the role of p70^S6K1^, an essential downstream signaling molecule of mTOR, in CXCL12-induced T cell migration both in vitro and in vivo. We have shown that the migration of human peripheral blood T cells mediated by CXCL12 can be blocked by rapamycin, an inhibitor of mTOR. Using stable T cell lines in which expression of p70^S6K1^ is knocked down, we were able to show a significant attenuation in migration in response to CXCL12, both in vitro and in vivo. Thus our data implicate a possible role for the mTOR pathway in CXCL12 mediated T cell signaling and migration.

## Results and Discussion

**Figure 1:**
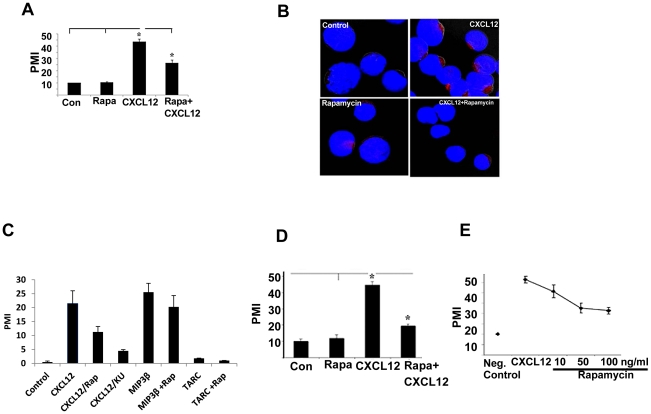
Rapamycin blocks CXCL12 induced migration and actin polymerization of T cells. (A) Primary human T cells were labeled with calcein (5 µM) for 1 h in media and washed. Pretreatment of labeled cells was done with or without rapamaycin (100 ng/ml) for 1 h. Cells (1×10^5^ in 100 µl) were placed in the upper wells of 24-well transwell migration chambers with 5 µm pores (Corning, Corning, NY). In the lower wells, either medium alone or CXCL12 (100 ng/ml) was added to a total volume of 600 µl, and the chambers were incubated for 2 hours at 37°C in 5% CO2 incubator. Triplicate well determinations were performed for each treatment. The level of fluorescence of cells migrating across the chamber was assessed using a microfluorimeter. * indicates the value is statistically significant at p<0.05 level (n = 4). PMI indicates ‘percent migration index’. (B) Primary human T cells were treated with CXCL12 for 30 minutes in the presence or absence of pretreated rapamycin. Confocal microscopy was done according to the procedure mentioned in Materials and Methods to monitor actin polymerization. Red color indicates the actin polymerization. Representative images are shown from each treatment group. (C) Effect of rapamycin and KU-0063794 (KU) on CXCL12-induced cell migration, and effect of MIP3β and TARC in the presence or absence of rapamycin on the migration of resting T cells were performed as described in panel A. (D) Migration assay for CEM cells was performed similarly as primary T cells described above. (E) Dose response curve for rapamycin effect.

To investigate the involvement of mTOR in CXCL12-induced T cell migration, human peripheral blood T cells were treated with CXCL12 for 2 hour in the presence or absence of rapamycin pretreatment, and the cell migration assay was performed using a transwell chamber. As shown in Fig. 1A, rapamycin pretreatment significantly inhibited CXCL12-induced T cell migration (43% inhibition). To test whether the rapamycin-mediated blockage in T cell migration was due to the inhibition of actin polymerization, CXCL12-treated cells in the presence or absence of rapamycin were stained with phalloidin, and the status of actin polymerization was examined by confocal microscopy. As shown in Fig. 1B, rapamycin pretreatment greatly reduced the actin polymerization induced by CXCL12 treatment. Recently, several mTOR specific inhibitors have been developed which are directed to the active site of mTOR, and thus inhibit both mTOR complex 1(mTORC1) and mTORC2: KU-0063794 [16], PP242 [17], and Torin [18]. We tested the effect of one of these inhibitors (KU-0063794) on CXCL12-induced T cell migration. As shown in Fig. 1C, KU-0063794 had a stronger effect than rapamycin, indicating the potency of the new generation mTOR specific inhibitor. We also tested the effect of several other chemokines on the migration of resting T cells and their sensitivities to rapamycin. As shown in Fig. 1C, MIP-3β (macrophage inflammatory protein-3beta) induced T cell migration, but this migration was insensitive to rapamycin, which is in agreement with the recently published data (Fig. 1C) [15]. Several other chemokines, TARC (thymus and activation-regulated chemokine), MIP-1α & β, MCP-2 (monocyte chemotactic protein-2) & 4, and MIG (monokine induced by gamma interferon), were also tested for T cell migration, but were found to be ineffective in resting T cell migration (Fig. 1C, and data not shown).

In order to investigate the role of mTOR in CXCL12-induced migration in more detail, we used the human T cell line CEM. As shown in Fig. 1D, CXCL12 promoted CEM chemotaxis and CXCL-12-induced migration of CEM cells was significantly inhibited by rapamycin pretreatment. The maximum inhibition (55%) was observed at 50 ng/ml concentration (Fig. 1E). Regarding the clinical relevance of the rapamycin inhibitory dose, serum concentrations of 2.7–27.1 ng/ml (2–10 mg orally/day) were attained in a phase I trial for patients with glioblastoma [19].

**Figure 2:**
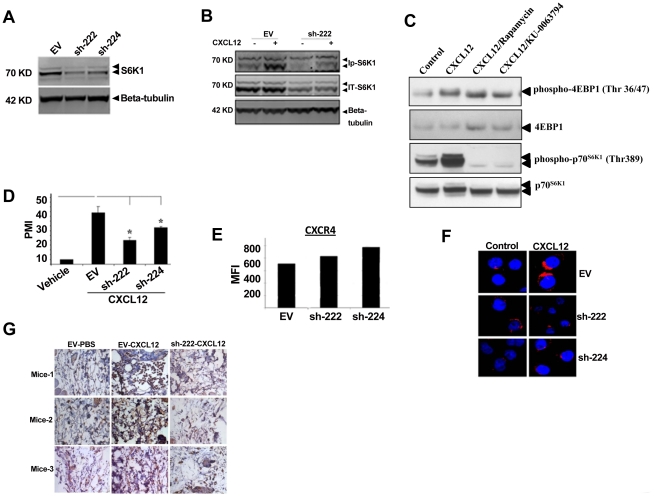
Role of mTOR in CXCL12-induced signaling and cell migration. (A) Whole cell lysates from CEM stable clones carrying either empty vector (EV) or shRNA constructs (sh-222 and sh-224) were analyzed by western blot analysis to determine the levels of total p70^S6K1^. (B) Whole cell lysates from EV and sh-222 clones treated with CXCL12 for 30 minutes were analyzed by western blot analysis to determine the levels of phospho-p70^S6K1^. (C) CEM cells were pretreated with rapamycin (100 ng/ml) and KU-0063794 (1 µM) for 1 hour, and then CXCL12 treatment was done for 30 minutes. Equal amounts of whole cell lysates were analyzed by western blot analysis. (D) Migration assay for EV, sh-222 and sh-224 clones were performed similarly as primary T cells described in Fig. 1A. (E) Surface expression of CXCR4 for EV, sh-222, and sh-224 were determined by FACS analysis, and the levels are expressed as mean fluorescence intensity (MFI). (F) Confocal microscopy to monitor CXCL12-induced actin polymerization in EV, sh-222, and sh-224 clones were performed similarly as primary T cells described in Fig. 1B. (G) In vivo migration of EV and sh-222 clone mediated by CXCL12 was determined as described in Materials and Methods. Tissue staining from individual mice is shown here.

Next, we developed p70^S6K1^ knock down clones of CEM cells by shRNA using a lentiviral gene transfer system. As shown in Fig. 2A, two independent knock down clones (sh-222 and sh-224) had about 50% reduced level of total p70^S6K1^ protein as compared to the clone carrying the empty vector (EV). CXCL12-induced phosphorylation of p70^S6K1^ was also reduced as shown in clone sh-222 (Fig. 2B). To determine the effect of CXCL12 on 4EBP1, another mTOR effector molecule, CEM cells were treated with either medium alone or with CXCL12 in the presence or absence of either rapamycin or KU-0063794 for 30 minutes, and whole cell lysates were analyzed by western blot analysis. As shown in Figure 2C, CXCL12 induced phosphorylation of both p70^S6K1^ (Thr 389) and 4EBP1 (Thr36/47), and both of these phosphorylations were blocked by rapamycin and KU-0063794. Cell migration assay showed significantly reduced levels of CXCL12-induced migration in sh-222 and sh-224 clones compared to the empty vector transfected clone (EV) (Fig. 2D). The inhibition in migration was not due to the down regulation of the cell surface expression of CXCR4, the receptor for CXCL12, as shown in Fig. 2E. Like peripheral blood T cells, CXCL12-induced actin polymerization was drastically reduced in sh-222 and sh-224 clones as compared to EV clone (Fig. 2F). To evaluate whether mTOR is involved in CXCL12-induced T cell migration in vivo, we used SCID mice to monitor the migration of clone sh-222 injected in the tail vein in response to CXCL12 applied in the flank region. Immunohistochemistry analysis of the skin tissue surrounding the site of injection using anti-CD4 antibody revealed significant migration of EV clone in response to CXCL12, which was drastically reduced in the case of clone sh-222 (Fig. 2G), indicating the involvement of mTOR signaling in migration of T cells in vivo against CXCL12.

It has been shown that CCL5-mediated T cell migration also involves mTOR through induction of protein translation of cyclin D1 and MMP-9 [9]. Since CCR5, receptor for CCL5, is only expressed in differentiated T cells (Th1 and Th2), CCL5 does not cause T cell chemotaxis in resting T cells [20]. On the contrary, CXCL12 and MIP-3β induced resting T cell migration, and only CXCL12, but not MIP3β, utilizes the mTOR pathway in T cell chemotaxis. The involvement of mTOR in CXCL12-mediated T cell migration is demonstrated not only by different mTOR specific inhibitors, but also by using p70^S6K1^ knock down cells, we were able to show a significant attenuation in T cell migration in response to CXCL12, both in vitro and in vivo. CXCL12 has been shown to activate AKT through PI3 kinase [5], [21]. AKT also controls mTORC1 by phosphorylating and inactivating tuberous sclerosis 2 (TSC2), the Rheb-GTPase-activating protein. Accumulation of Rheb-GTP upon AKT activation results in the activation of mTORC1 [22], [23]. Besides cell migration, CXCL12 is also involved in multiple different processes such as immune regulation, inflammatory responses, and cancer development [24]. These processes require plenty of energy. On the other hand, mTOR is a nutrient sensor and a master regulator of translational machinery [25]. So it is not surprising that CXCL12 activates the mTOR pathway to promote anabolic processes. Collectively, our observations give insight into the future perspective regarding the possibility of the clinical usage of rapamycin as a blocker of CXCL12 induced T cell migration and inflammatory diseases.

## Materials and Methods

### Cell Culture

Peripheral blood mononuclear cells (PBMC) were collected from healthy donors who provided informed written consent. The protocol (ID: 2003-054) for collecting blood from normal donors was approved by the Institutional Review Board in the National Institute on Aging at the National Institutes of Health. PBMCs were isolated by Ficoll-Paque density gradient centrifugation. Total resting T cells were purified from PBMC using MACS Pan T cell isolation kit II (Miltenyi Biotec). CEM, a human T-cell leukemia cell line[26], and purified T cells were cultured in RPMI 1640 with 10% FBS, 100 U/ml penicillin, 100 µg/ml streptomycin, and 2 mM glutamine. Cells were treated with CXCL12 (100 ng/ml) for 30 minutes in the presence or absence of rapamycin (100 ng/ml) pretreated for 1 hour.

### Reagents

Recombinant CXCL12 was purchased from Peprotech (Rocky Hills, NJ). Rapamycin was purchased from Calbiochem (San Diego, CA). Fluorescence calcein was purchased from Invitrogen (Carlsbad, CA). Anti-p70^S6K1^, anti-4EBP1, and anti-phospho-4EBP1 were purchased from Cell Signaling Technology (Beverly, MA). Anti-phospho-p70^S6K1^ (Thr 389) was purchased from R&D Systems (Minneapolis, MN). shRNA constructs against p70^S6K1^ (RHS4531-NM-003161) and pGIPZ empty vector were purchased from Open Biosystems (Huntsville, AL). Transwell chemotaxis chambers were purchased from Corning Life Sciences (Pittsburgh, PA).

### Chemotaxis Assay

T cell migration was performed in transwell chemotaxis chambers according to the protocol described earlier [27]. Cell migration is expressed in percent migration index (PMI).

### shRNA Stable Transfectants

shRNA plasmid (V2LHS_153622) targeting p70^S6K1^, and the appropriate empty vector, pGIPZ, were transfected into the CEM cell line according to the method described earlier [28]. Stable transfectants were selected by the addition of 2.0 µg/ml of puromycin followed by single cell cloning.

### Confocal Microscopy

Cells were fixed in ice cold 90% methanol in PBS for 20 minutes followed by staining for polymerized actin using phalloidin-labeled Alexa Fluor- 594 (Invitrogen, Carlsbad, CA). Cells were blocked with 5% BSA solution in PBS and then stained with conjugated phalloidin overnight at 4°C. Unbound fluorophores were washed with ice cold PBS mixed with 0.1% Tween-20. Cells were subsequently cytospun on a coated slide (Fisher, Pittsburgh, PA) and then mounted with mounting media (Prolong Gold, Invitrogen, Carlsbad, CA). Acquisition of images was performed using a Carl Zeiss Axiovert S100 microscope (Carl Zeiss, Thornwood, NY). Images were deconvoluted with software Zeiss lsm 510.

### In Vivo Migration Assay

For animal study, the protocol (Protocol Number: 272-LI-2010) was approved by the Animal Care and Use Committee in the National Institute on Aging at the National Institutes of Health. Equal numbers of CEM cells from either a p70^S6K1^ knock down clone or a vector containing clone were injected into the tail vein of severe combined immunodeficiency mice (SCID). Simultaneously, PBS or CXCL12 (1 µg in 50 µl) was injected subcutaneously to the right anterior flanking region. After 18 h, mice were sacrificed followed by isolation of skin tissue around the point of injection. Tissues were fixed in 4% ice-cold paraformaldehyde overnight and were embedded in paraffin. Paraffin sections were then deparaffinized and blocked as previously described [29], followed by an overnight incubation at 4°C in 10 µg/ml anti-human CD4 antibody. Tissues were then probed with biotinylated secondary Ab (VECTOR Laboratory) and Streptavidin-HRP (Thermo Scientific), and developed using DAB plus substrate system (Thermos Scientific). All slides were also counter-stained with hematoxylin.
